# Editorials

**Published:** 1898-04

**Authors:** 


### HOMŒOPATHIC PHYSICIAN,

A MONTHLY JOURNAL OF

HOMŒOPATHIC MATERIA MEDICA AND CLINICAL MEDICINE.

"If our school ever gives up the strict inductive method of Hahnemann, we are lost, and deserve only to be mentioned as a caricature in the history of medicine."—CONSTANTINE HERING.

Vol. XVIII. APRIL, 1898. No. 4.

### EDITORIALS.

NOTICE TO THE PROFESSION.—The arrangement heretofore existing by which Alfred Heath & Co. have been our European agents, having been terminated, all foreign subscribers will, hereafter, please communicate directly with the home office, 1231 Locust Street, Phila., Pa., U. S. A.

---

SILICEA.—The great characteristic of Silicea, the keynote of Dr. Guernsey, is difficult hard stool, the fæces are large, and when partly expelled slip back again. Alumina has hard stool and small, but cannot be expelled. It causes great straining. Causticum, the stool is large, hard, and coated with mucus. It also provokes severe straining, with redness and perspiration of the face. This is another of Dr. Guernsey's keynotes. Silicea has very fœtid smelling stools. Calcarea-carb., the stools smell sour.

Silicea has sediment of red or yellow sand. Lycopodium has red sand in the urine. It is Dr. Guernsey's keynote for Lycopodium. Sarsaparilla has sediment of white sand.

Silicea has menses too early and too scanty. Calcarea-carb. has menstruation too early and too profuse. This is Dr. Guernsey's keynote.

Silicea has menses too profuse, but at the same time, *too late*.

Silicea is useful in pregnancy where there are painful motions of the fœtus. Opium has also movements of fœtus.

Silicea has stitches in chest and sides of chest through to the back.

Mercurius has stitches in the middle of the chest, in the region of the sternum through to the back.

Stitches in upper left lobe of lungs through to the back indicate Phosphorus. If the stitches are in the left lower lobe Sulphur is the remedy. If the stitches are in the *right upper* lobe, Borax; if in *right lower* lobe, Bryonia. All these are keynotes of Dr. Guernsey. They have been given before in this journal, but need to be repeated. In addition to them the Editor on his own account gives the following:

*Chest Pains.*—Dull pain in left pectoralis major, extending to the muscles of the shoulder and point of the shoulder-blade, and through lower part of the arm to the elbow, better throwing the arm back, striking upon it, and lifting shoulder, Arsenicum-metallicum.

Sticking pain in right side of chest at fifth rib, worse on moving body or arm, extending to upper part of the zyphoid cartilage and impeding breathing, Apis.

Pains in chest under *right* nipple in pleuro-pneumonia, Ranunculus-bulbosus.

Pain under *left* nipple, Comocladia.

Pain in *left* upper chest at collar-bone, Myrtis-communis.

Pain in *right* upper chest at collar-bone, Illicium-anisatum.

In the *right* chest dull pain in circumscribed spot, worse on inspiration, Kali-bichromicum.

In *right* chest, sticking pains, Nitric-acid.

In *right* chest at third rib severe pain, Illicium-anisatum. (See preceding page, third line from the bottom.)

In *left* chest at third rib severe pain, Pix-liquida.

In *left* chest shooting or stitching pain going to shoulder-blade, Calcarea-phosphorica.

In *left* chest, beating in a small spot, Calc-phos.

In *right* side of chest between eighth and ninth ribs, and in whole right side from spine to sternum, stinging, tearing pains in the intercostal spaces, worse from four to seven o'clock P. M., producing anxiety and dyspnœa, Chinium-sulphuricum.

Can't bear pressure of the clothing on chest, Causticum.

Chest sensitive to a draft of air; it gives fresh cold, Phosphoric-acid.

Silicea has curvature of the vertebræ. Calcarea-carb. is a great remedy for this disease, and so also are Fluoric-acid and Baryta-carb. The individual symptoms in each case must decide.

Silicea has glandular swellings in the neck and arm-pits, with suppuration. Calcarea has hard swelling of these glands without suppuration.

Silicea has heaviness and paralytic weakness of the forearms. Calcarea has weakness of fingers; they will not hold anything.

Silicea patient drops things by reason of this weakness. This idea of dropping things from the hands suggests a number of remedies.

Hellebore is the principal remedy in this regard. The indication is that if the attention is diverted from the act, the object falls from the hands.

Apis and Bovista both have dropping things from awkwardness.

Weakness of wrist causing the hand to drop things indicates Kali-carb.

